# The Relation between Alpha/Beta Oscillations and the Encoding of Sentence induced Contextual Information

**DOI:** 10.1038/s41598-019-56600-x

**Published:** 2019-12-27

**Authors:** René Terporten, Jan-Mathijs Schoffelen, Bohan Dai, Peter Hagoort, Anne Kösem

**Affiliations:** 1 Max Planck Institute for Psycholinguistics, Wundtlaan 1, 6525 XD Nijmegen, The Netherlands; 2 Donders Centre for Cognitive Neuroimaging, Kapittelweg 29, 6525 EN Nijmegen, The Netherlands; 3 Lyon Neuroscience Research Center (CRNL), Brain Dynamics and Cognition Team, INSERM U1028, CNRS UMR5292, Université Claude Bernard Lyon 1, UdL, Lyon, France

**Keywords:** Electroencephalography - EEG, Cognitive neuroscience

## Abstract

Pre-stimulus alpha (8–12 Hz) and beta (16–20 Hz) oscillations have been frequently linked to the prediction of upcoming sensory input. Do these frequency bands serve as a neural marker of linguistic prediction as well? We hypothesized that if pre-stimulus alpha and beta oscillations index language predictions, their power should monotonically relate to the degree of predictability of incoming words based on past context. We expected that the more predictable the last word of a sentence, the stronger the alpha and beta power modulation. To test this, we measured neural responses with magnetoencephalography of healthy individuals during exposure to a set of linguistically matched sentences featuring three levels of sentence context constraint (high, medium and low constraint). We observed fluctuations in alpha and beta power before last word onset, and modulations in M400 amplitude after last word onset. The M400 amplitude was monotonically related to the degree of context constraint, with a high constraining context resulting in the strongest amplitude decrease. In contrast, pre-stimulus alpha and beta power decreased more strongly for intermediate constraints, followed by high and low constraints. Therefore, unlike the M400, pre-stimulus alpha and beta dynamics were not indexing the degree of word predictability from sentence context.

## Introduction

Sentence level language comprehension results from dynamic cognitive processes which combine and unify smaller linguistic units to create meaning^1–6^. These cognitive processes occur online, while the sentence unfolds, instantiating unified meaning which relates to the computation of semantics, spanning the whole utterance. During this process, a context representation is compared and updated on a moment to moment basis. The bias provided by the momentarily established context alters subsequent linguistic processing^7–9^. One classical approach to investigate the impact of linguistic predictions at the neuronal level is to measure the N400 component, which is called the M400 in magnetoencephalographic (MEG) studies^10–12^. The N400 functionally marks how surprising the occurrence of a target word is provided the past sentential context^13^. The N400 amplitude is stronger for anomalous or unexpected items, but importantly it is also influenced by how constraining the preceding sentential context is (i.e. how predictive are subsequent linguistic items based on past context), such that the N400 amplitude increases with lower sentence context constraints^11,14–18^. While not being linked to predictive processes directly, these N400 modulations suggests that sentence context constraints alter predictions that are encoded *prior* to target word occurrence.

Prediction in its minimal sense can be understood as changes in brain *states* in response to contextual information which facilitate the processing of new input^19,20^. Recent evidence suggests that neural rhythmic activity could be involved in the prediction of linguistic input during sentence processing. Neural oscillatory responses have been linked to the N400 and semantic predictions, such as theta (4–7 Hz) oscillations^21,22^ and gamma band (>40 Hz) activity^23–26^. Importantly however, theta and gamma oscillatory activity has mostly been observed as neural markers for semantic predictions after target word presentation. In contrast, and to the interest of the present study, other brain oscillatory responses in the alpha (8–12 Hz) and beta (16–20 Hz) frequency ranges have been linked to both domain-general and linguistic predictive mechanisms *prior* to the apparition of a target word. Alpha and beta oscillations are hypothesized to reflect a domain-general mechanism for the prediction of upcoming sensory input^27,28^ and to constitute top-down mechanisms that shape the communication of sensory information between distant neural networks^29–31^. Beyond sensory processing, recent theory and evidence suggests that alpha and beta oscillations would also be involved in linguistic prediction^19,23,28^. Decreases in alpha power^21,25,32,33^ and low-beta power^25,33,34^ have previously been linked to the processing of sentential context constraints. Specifically, the power decrease has been found to be stronger when sentential context is highly predictive of the last word of the sentence than when the prediction of the last word cannot be made based on past context^21,25,32–37^. The power decrease has been explained to reflect stronger engagement of the brain areas of the language network in scenarios in which predictions can be formed from past sentential context^25,38^. Yet, the evidence for alpha and beta oscillations being involved in language prediction is still debated, as it has only been observed between extreme situations, i.e. between very predictable sentences or completely unpredictable sentences^21,25,33^. If alpha and beta power reflect the degree of predictability of an upcoming word, we hypothesized that alpha/beta power should gradually decrease with higher context constraint.

**Figure 1:**
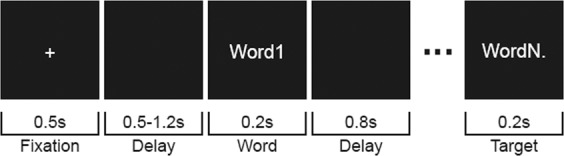
A schematic display of a trial procedure. A trial began with the display of a fixation period, followed by a blank screen. Subsequently the sentence was visually displayed by a word by word presentation, up to the final word as indexed by the period. Between words, a black screen served as delay before a subsequent word was shown.

To test this, we presented multiple graded groups of sentences context constraints. Participants passively read sentences belonging to either a low (LC), medium (MC) or high (HC) context constraining condition (Table 1). Crucially, we constructed the sentence material using triplets of sentences, and modulating the context constrain by only changing one word in the sentence (see Methods for details). Neuronal activity was measured online using MEG, before and after display of a target word. Based on previous findings^21,25^, we predicted that pre-stimulus alpha and beta power would differ between different conditions of predictability. The power decrease is expected to be strongest for the HC, followed by the MC and LC condition (Fig. 1).

**Table 1:** Example Dutch sentence triplet from the final stimulus set with its English translation.

Stimulus material examples	
Condition	Stimulus
**HC**	(NL) Op dit *gebouw* heb je een goed uitzicht.
	(EN) On this *building* you got a good view.
**MC**	(NL) Op deze *toren* heb je een goed uitzicht.
	(EN) On this *tower* you got a good view.
**LC**	(NL) In deze *wijk* heb je een goed uitzicht.
	(EN) In this *area* you got a good view.

The context constraining conditions were manipulated by changing one context constraining word.

## Materials & Methods

### Participants

In total, thirty-five students (mean age 24 years, range 18–43; 16 males) took part in the experiment. All participants provided their informed consent in accordance with the declaration of Helsinki, and the local ethics committee (CMO region Arnhem-Nijmegen). All experimental protocols were approved by the review board of the Donders Centre for Cognitive Neuroimaging prior to the start of the experiment. The participants were all Dutch native speakers, right-handed, had normal or corrected-to-normal vision and none of them suffered from neurological impairment or dyslexia. Two participants were excluded because they did not finish the experiment. Therefore, thirty-three participants were included for further analyses (mean age 24 years, range 18–43; 15 males).

### Stimulus material

The stimulus set consisted of 253 sentence triplets, including 203 critical and 50 filler sentence triplets. Each sentence within a critical triplet belonged to either a high context (HC), medium context (MC), or low context (LC) constraining condition. The different degree of constraint within a triplet was achieved by manipulating only one word, the *context constraining word*, which was always at the same position within a sentence with regard to a triplet (Table 1). Across the conditions, these context constraining words were matched with regard to word length (F(2, 606) = 0.784, p = 0.457, with a Mean (SE) of HC: 7.12 (2.26); MC: 7.1 (2.54); LC: 7.37 (2.61)) and word frequency (F(2, 584) = 1.984, p = 0.138, with Mean (SE) of HC: 2.4 (0.78); MC: 2.56 (0.87); LC: 2.5 (0.84); based on the Dutch SUBTLEX-NL database^40^. The stimuli were pretested in a sentence completion task in order to verify the step-like degree of context constraints within a triplet (from high, to medium, to low). For this task - independent from the MEG experiment - a sample of participants (N = 51) were required to complete a sentence presented on a computer screen, for which the final word was missing. Participants performed the experiment with one of three counterbalanced lists. Each list included the same number of critical sentences from either of the three context constraining conditions. The validation of the conditions was performed in two subsequent steps: first, the degree of context constraint per sentence was evaluated by calculating the percentage of participants that would finish a sentence with the same word. These probabilities are thought to reflect the degree to which the sentential context biases subsequent language processing. As expected, HC sentences resulted in the highest percentage of participants proposing the same word as cloze (Mean (SE) = 77% (17.74)), followed by MC (Mean (SE) = 50% (18.67)) and LC (Mean (SE) = 28% (11.97)). The three conditions differed significantly from each other with regard to their degree of context constraints (F(2, 606) = 442.842, p < 0.001).

Second, and in order to create the final stimulus set, the final word from the HC sentences with the highest percentage was chosen as sentence ending for all sentences within a triplet. This approach resulted in cloze probabilities for the final word - the *target word* - that were different from the percentages of the context constraints for the MC and LC conditions. Still, the cloze probabilities differed significantly between conditions (F(2, 606) = 468.155, p < 0.001), with HC showing the highest cloze probability (Mean (SE) = 77% (17.74)), followed by MC (Mean (SE) = 42% (25.94)) and LC (Mean (SE) = 15% (15.82)). The cloze probabilities are thought to reflect how surprising the final word occurrence is, given the past sentential context. In our stimulus set, context constraint measures were highly correlated with measures of cloze probability (r = 0.93, p < 0.001).

In the MEG experiment, participants were presented with one of the counterbalanced lists, with an additional set of 50 filler sentences. The filler sentences did not differ between lists but followed a different sentence structure as compared to the critical sentences.

### Experimental procedure

Participants were comfortably seated in a dimly illuminated and magnetically shielded room. All participants were instructed to place their arms on the arm rest of the chair, with access to a button box with their right hand. In front of each participant, at a distance of 80 cm and with a 25°–35° viewing angle, a screen was located on which all stimulus material was displayed. The words were shown in black, on a grey background. Participants were instructed to silently read the displayed sentences on the screen, and to focus on the content of each sentence. Furthermore, it was highlighted that sometimes (after 20% of the sentences; subjects were not informed about the precise percentage) a question would be asked about the content of the previous displayed sentence. The participants were then required to answer this question with ‘yes’ or ‘no’ by button press. The answer possibilities (‘yes’/‘no’) were randomly displayed on the left or right side of the screen and a left or right button had to be pressed accordingly. These question trials were catch trials, intended to ensure that participants were actively processing the meaning of the sentences, without focusing their attention on the contextual constraints. A trial started with the display of a fixation cross in the middle of the screen for 500 ms. The fixation cross was followed by a blank screen for a random interval of 500–1200 ms. Subsequently, the word-by-word presentation of the sentence began, with each word being displayed for 200 ms, followed by a blank screen of 800 ms. An interval of 1000 ms was chosen in order to record pre-stimulus alpha and beta activity that is not influenced by the evoked response to the previous displayed word. After a sentence ended, another blank screen occurred for 2000 ms (Fig. 1). After that, either a catch question was displayed, with the whole question in the middle of the screen and the yes-no answers randomly split to the left or right side, or the next trial began. In total, participants read 253 sentences (253 trials) in random order, which came from one of three lists, counterbalanced on the three levels of context constraints. The total amount of trials was divided into four blocks, separated by small breaks in-between. The length of a break was self-determined by the participants and the task could be continued by button press. In total, the data acquisition lasted 60 min.

### Data acquisition

All data were acquired using a 275 axial gradiometers CTF Omega MEG system. Horizontal and vertical bipolar electrooculography (EOG) as well as electrocardiography (ECG) were recorded in order to discard eye blinks, eye movements and heart beats contaminated trials. All electrophysiological signals were low-pass filtered at 300 Hz, digitized at 1200 Hz, and stored for off-line analysis. Three coils were placed on the nasion and the left and right ear canal to register the head position with respect to the gradiometers. The coils enabled real-time monitoring of the head position throughout the experiment^41^. Next to the MEG recordings, magnetic resonance images (MRIs) were obtained from 32 of the participants with a 1.5 T or 3.0 T Siemens system. By means of attached markers at the same anatomical locations as the head coils, the MRIs could be aligned to the MEG coordinate system.

### Data preprocessing

All data were analyzed using the open-source Matlab toolbox Fieldtrip^42^. From the MEG data, a time-window of interest was segmented 2 s before and after the onset of a sentence’s final word for each trial. This segmentation therefore included the blank delay period just before onset of the target word, where the effect of context constraints is expected to occur, and the period after onset of the target word. The segmented data were low-pass filtered at 150 Hz. The 50 Hz line noise components were removed by using a notch filter. Artifact identification and rejection was done in three steps. First, MEG jump and muscle artifacts were identified by visual inspection of amplitude variance over trials. Second, artifacts related to eye-movements and cardiac activity were identified and removed by means of an independent component analysis (fastICA)^43^, followed by backprojection. The independent components were visually inspected and removed from the sensor data, if they resembled heartbeat, eye-movements or blinks (as compared to the recorded EOG and ECG). Third, the resulting data were again visually inspected to remove any remaining artifacts. From this procedure, on average 11% of trials and 1.5% of MEG sensors were excluded from further analysis.

### Event-Related Field (ERF) analysis

Event-related fields were investigated to observe M400 modulations after the last word onset. This correlate is the magnetic counterpart of the classical N400 measured by electroencephalography and inhabits the same time-course and response properties^44,45^. For each condition, the epochs were first low-pass filtered at 35 Hz. All ERFs were then baseline corrected based on a time window of −300 ms–0 ms relative to target word onset. To facilitate comparison across participants the ERFs were transformed to a combined synthetic planar gradient representation^46^. The M400 component was calculated by averaging over the planar gradient field amplitude from 250 ms to 600 ms following target word onset.

### Time-frequency analysis

Time-frequency analysis was first done for the time-window of −800 ms to 0 ms relative to the sentence’s target word onset, including only the blank delay period. Additionally, the M400 sensitive time-window after target word onset was considered for time-frequency analyses, including a window from 200 ms up to 700 ms. Alpha and beta power were estimated for each condition using fast Fourier transform for a frequency range of 8 Hz to 12 Hz for the alpha, and 16 Hz to 20 Hz for the beta frequency bands (based on Wang *et al*.^25^), with a Hanning-tapered 500 ms sliding window in time steps of 10 ms. No baseline correction was performed on the time-frequency data, statistics were performed by contrasting individual conditions of context constraints. Time-frequency plots were created using a wider frequency range of 2 Hz to 30 Hz (using a fixed 500 ms sliding Hanning window in time steps of 10 ms and frequency steps of 2 Hz) for a time window of −1000 ms to 1000 ms. Power was averaged over channel clusters that were a result of the statistical comparison between conditions for the alpha and beta frequency band respectively.

### Source analysis

To estimate the sources of the oscillatory activity, the Dynamical Imaging of Coherent Sources (DICS) beamforming approach was applied to the data^47^. The volume conduction model was constructed from the individual anatomical MRI as a single shell representation of the inside of the skull. This model was used to compute the forward model according to Nolte^48^. The initial co-registration between the headmodel and MEG sensors was achieved by manually identifying the anatomical landmarks of the nasion and two auricular fiducials, and was additionally refined, using the subject-specific three-dimensional digitised representation of the scalp, as obtained by a Polhemus digitizer. The source space was discretized into a three dimensional grid with a 6 mm resolution. Source reconstruction was performed using a spatial filter, which was computed by combining the cross-spectral density (CSD) matrices from all three conditions (HC, MC, LC). The CSDs were computed using the Fast Fourier transform of the data with multitapering, with a center frequency of 10 Hz or 18 Hz for the alpha (averaged over the time window 540–0 ms, relative to target word onset) and beta (averaged over 450–0 ms) frequency band respectively. All visualizations are based on interpolated data onto the MNI template. The different conditions of the source reconstructed data were compared based on cluster-based permutation statistics as described below.

### Cluster-Based permutation statistics

Statistical evaluation was done using non-parametric cluster-based permutation tests^49^. First, we computed F-statistics to quantify the effect of context constraints (three levels: HC, MC, LC) for each sensor and time point. These F-statistics were used to define the clusters for the non-parametric statistical testing: clusters consisted of samples whose F-values were above threshold (threshold: F-value associated with a p-value of 5%) and were adjacent in space and time. Cluster-level statistics were computed by taking the sum of F-values within each cluster. The distribution of the cluster-level statistics under the null hypothesis was obtained by repeating this procedure for 5000 permutations of random relabeling of the conditions. Clusters whose test-statistics fell in the highest 5th percentile of its reference distribution were considered significant.

## Results

### Behavioral performance

In order to confirm the participant’s attention to the experimental task, their performance was measured during catch trials that occurred after presentation of 20% of the sentences. The overall accuracy measures show a mean ceiling performance of 95% (SE = 4.65), 96% (SE = 4.42) and 97% (SE = 2.83) for the HC, MC and LC sentences respectively. There were no significant differences in accuracy with respect to the different conditions (Accuracy: F(2, 31) = 0.474, p = 0.627). This indicates that the participants were paying attention to the content of the presented sentences.

### Event-Related fields after target word onset

**Figure 2:**
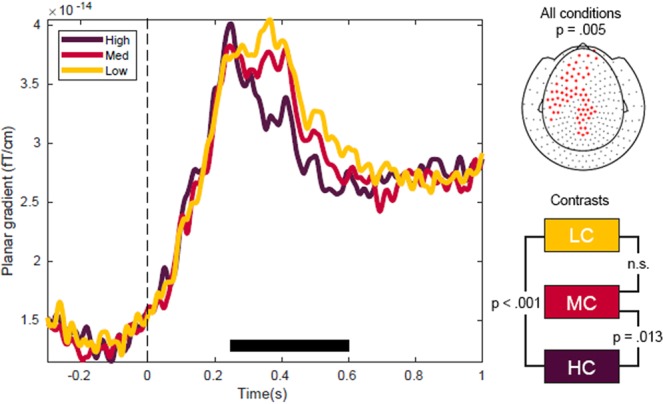
The event related fields of the M400 component at target word onset as averaged across channel clusters. The M400 amplitude is gradually modulated by the degree of context constraints, resulting in the lowest amplitude in HC, followed by MC and LC. The black horizontal line in the main figure highlights the time window [250 ms, 600 ms] pre-defined for the spatial cluster-based permutation test. The upper right topography shows the MEG sensors that formed a cluster as result from the statistical comparison of the conditions. The effect is most pronounced over left lateralized sensors.

In this experiment, participants read words preceded by a context with different degrees of constraint (three context-constraint conditions: high, medium and low context constraints) while brain signals were recorded online. We first analyzed the effect of context constraints on the event-related fields upon target word presentation. Based on previous literature^11,14^ we expected a monotonous relationship between cloze probability and the M400 component. Consistently, as can be seen from the amplitude fluctuations of the event-related activity (Fig. 2), amplitude differences between the three conditions emerged within the typical M400 time-window. The M400 amplitude strength decreased with increasing cloze probability, such that the HC condition displayed the lowest M400 amplitude, followed by the MC and LC conditions. The cluster-based statistics revealed a main effect of context constraints on the M400 amplitude strength in a pre-defined time window of 250 ms to 600 ms after target word onset; this effect was most pronounced over a left-frontal localized cluster of sensors (Fig. 2, cluster *p* = 0.005). The post-hoc contrasts (based on pairwise T-tests) revealed that the effect was mainly driven by a difference between HC vs. LC (*p* < 0.001) and HC vs. MC (*p* = 0.013). Although the M400 amplitude was smaller for MC than for LC condition, this difference was not significant. These effects were overall in line with the current literature showing that the M400 amplitude reflects semantic retrieval and unification of the target word with the preceding context.

### Alpha/Beta power modulations before target word onset

**Figure 3:**
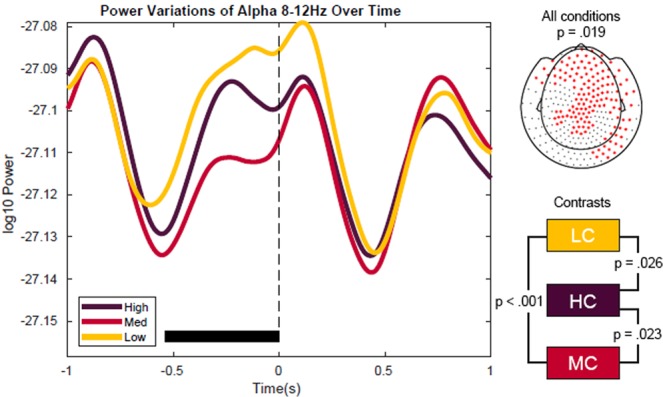
Alpha power fluctuations vary as a function of context constraint before target word onset as averaged across channel clusters. The black horizontal line in the main figure highlights the time window [−540 ms, 0 ms] of the spatio-temporal cluster for which the difference between conditions was significant. The upper right topography shows the MEG sensors of the cluster. The effect of context constrains is most pronounced over frontal and posterior sensors. Over these sensors, alpha power showed the strongest decrease for the MC condition, followed by HC and LC.

**Figure 4:**
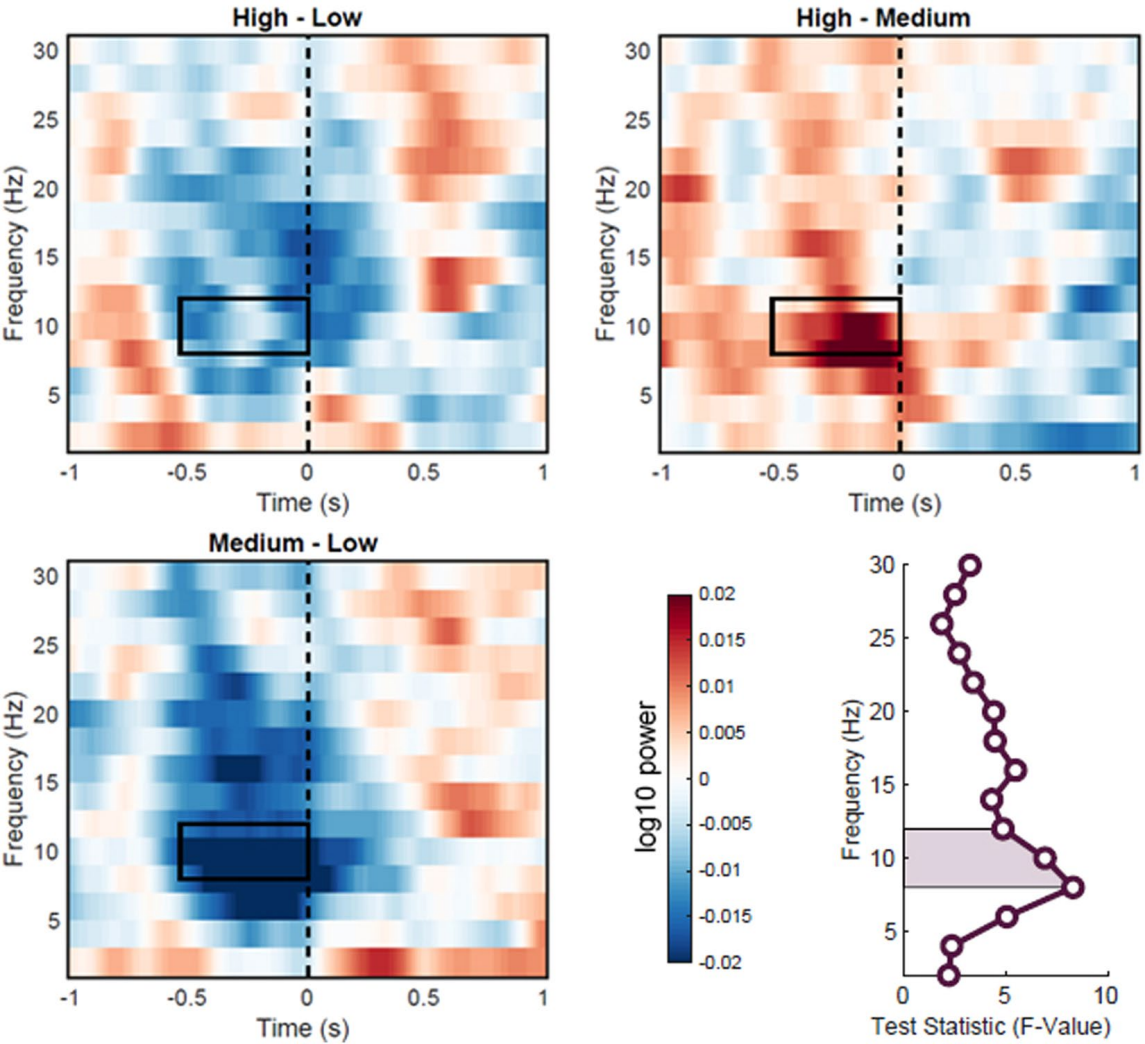
Time-frequency power contrasts between context constraint conditions within the alpha band sensor cluster. Time-frequency plots indicate power as averaged across sensors that were identified by the cluster-based permutation statistics of the alpha frequency band (8–12 Hz). The dashed line marks the onset of the target word. The rectangle indicates the time-window of the respective cluster, and alpha frequency band. Lower right figure shows the F statistics of the main effect of context constraints across frequencies, averaged within the time window [−540 ms, 0 ms]. The area under the curve indicates the pre-defined alpha frequency range used for cluster based analysis (8–12 Hz). The effect of context constraint is stronger in the lower alpha range (8 Hz).

Next, we investigated the effect of content constraints on alpha and beta power modulations before target word onset. Based on earlier research^25^, an effect was suggested to occur during the delay period, just before the display of the target word. The cluster-based statistics for the alpha (8–12 Hz) frequency band revealed a significant difference between all three conditions with regard to power (*p* = 0.019) for a time-window of −540 ms to 0 ms relative to target word onset. This effect was most pronounced over a widespread set of sensors, including anterior, central and posterior sensors (Fig. 3). Over these sensors, alpha power showed the strongest decrease for the MC condition, followed by HC and LC (Fig. 3). The post-hoc contrasts of these conditions indicate that the power decrease is significantly different between HC vs. MC (*p* = 0.023), MC vs. LC (*p* < 0.001), and HC vs. LC. (*p* = 0.026). Post-hoc comparisons of power (averaged over t(alpha) = −540–0 ms and t(beta) = −450 ms−0 ms) with respect to the whole spectrum (2 Hz to 30 Hz) showed that the strongest difference between context constraints peaks in the lower alpha range (8 Hz, Fig. 4).

**Figure 5:**
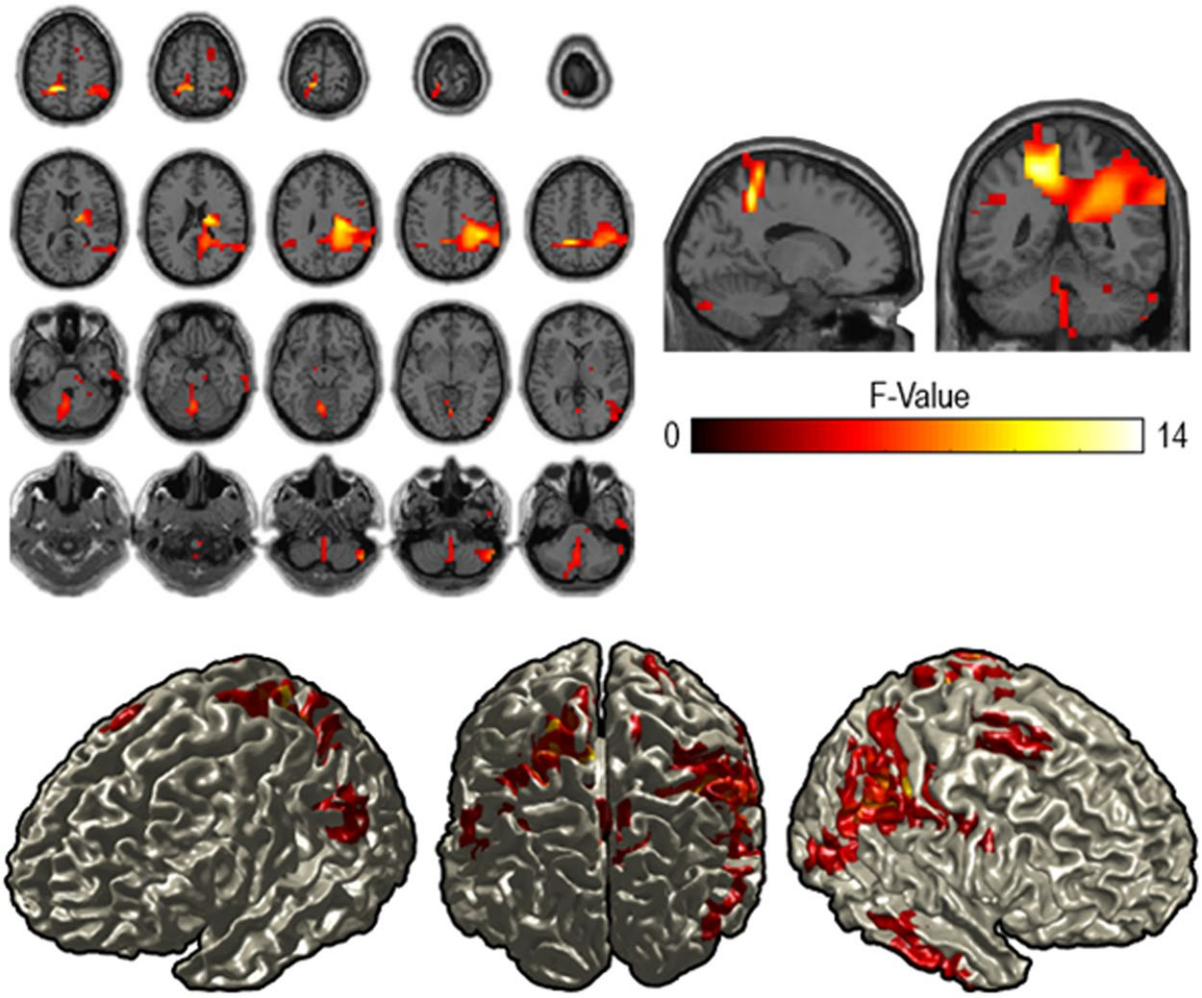
Reconstruction of the effect of context constraints in the alpha range (8–12 Hz). The source statistics reveal that the effect of the context constraint manipulation is most pronounced over left and right parietal regions. Upper figures show horizontal and sagittal as well as coronal slices for the alpha frequency band. Lower figures represent surface plots of the source statistics (F-values are thresholded at p < 0.05 and controlled for multiple comparisons using cluster-based permutation tests).

We performed source reconstruction to allow for a more detailed description of the brain areas involved in the observed sensor-level effect. Source-level statistical evaluation indicated that this effect was most pronounced in parietal areas, with a bias to the right hemisphere (Fig. 5, p = 0.003, cluster-based corrected).

**Figure 6:**
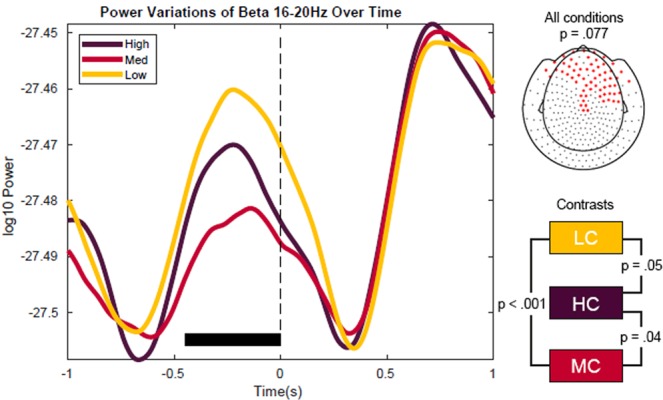
Beta power fluctuations as a function of context constraints as averaged across channel clusters. The striped line marks target word onset. The black horizontal bar indicates the time window of the cluster [−500 ms, 0 ms]. The right topography shows the sensors of the cluster. The effect of context constrains (which does not reach significance threshold) is most pronounced over frontal sensors. Over these sensors, beta power showed the strongest decrease for the MC condition, followed by HC and LC.

**Figure 7:**
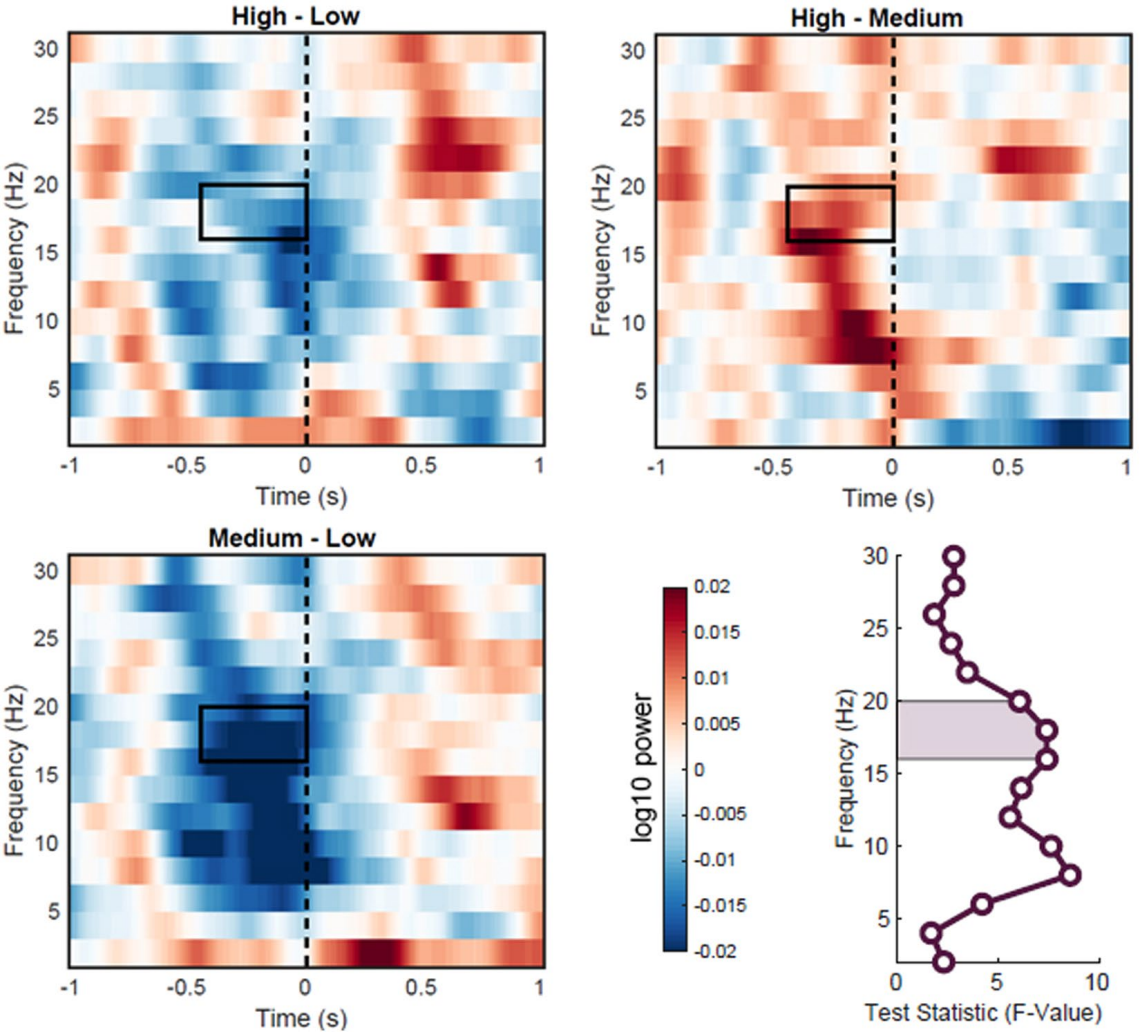
Time-frequency power contrasts between context constraint conditions within the beta band sensor cluster. Time-frequency plots are averaged across sensors that were identified by the cluster-based permutation statistics of the beta frequency band (16–20 Hz). The dashed line marks the onset of the target word. The rectangle indicates the time-window of the respective cluster, and the beta frequency band. Lower right figure shows the F statistics of the main effect of context constraints across frequencies, averaged within the time window [−500 ms, 0 ms]. The area under the curve shows the pre-defined beta frequency range used for cluster-based analysis (16–20 Hz). For this cluster (over frontal sensors) the effect of context constraint is strongest in the beta range (16–18 Hz) and in the lower alpha range (8–10 Hz).

The sensor-level analyses in the beta (16–20 Hz) frequency band revealed a similar tendency as for the alpha results, though the effects were not significant (cluster with lowest p-value in cluster-based corrected statistics: p = 0.077, see Figs. 6 and 7).

**Figure 8:**
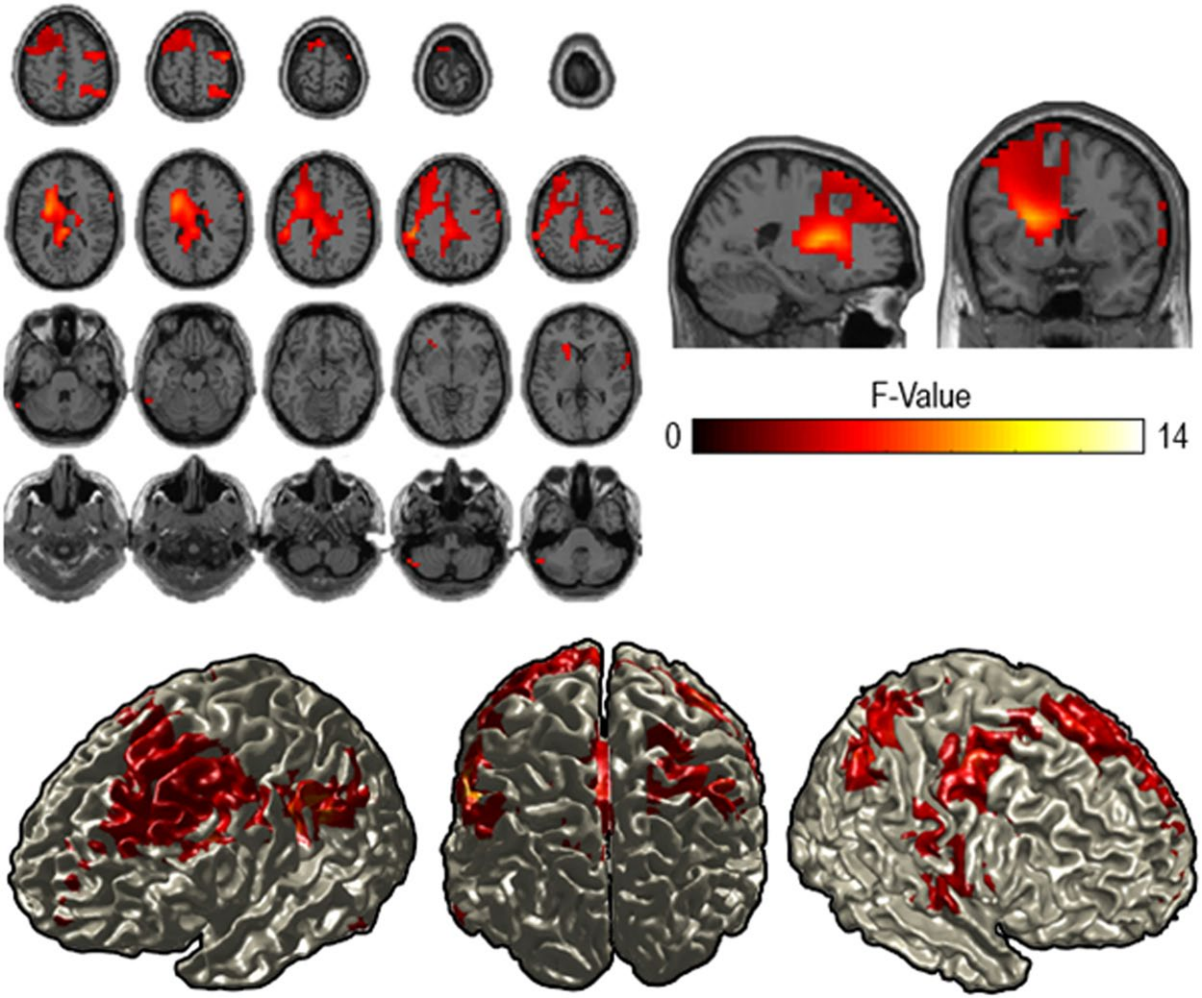
Reconstruction of the effect of context constraints in the beta range (16–20 Hz). The source statistics reveal that the effect of the context constraint manipulation is most pronounced over left and right dorsolateral prefrontal regions and parietal regions. Upper figures show horizontal and sagittal as well as coronal slices for the beta frequency band. Lower figures represent surface plots of the source statistics (F-values are thresholded at p < 0.05 and controlled for multiple comparisons using cluster-based permutation tests).

Source statistics in turn indicated a significant F-contrast across all conditions with the effect being most pronounced over a set of frontal and parietal areas, biased to left frontal cortex (Fig. 8, p = 0.002, cluster-based corrected). The power fluctuations were, similar to the results on alpha power, non-monotonically related to each other. The MC condition was again displaying the strongest decrease followed by HC and then the LC condition (Figs. 6, 7).

### Alpha/Beta power modulations after target word onset

To cover any oscillatory effects within the M400 sensitive time-window, alpha and beta power modulations were investigated as a function of context constraints after target word onset. The individual cluster-based statistics for both frequency bands revealed no significant difference between the three conditions with regard to power, within this particular time-window.

## Discussion

The current study investigated the role of pre-stimulus alpha and beta oscillations as a neural marker for sentence context constraints. Our results confirm the sensitivity of pre-stimulus alpha and beta power to different levels of constraint. Power of alpha and beta decreases when sentential context constraints are high as compared to when the context constraints are low, in line with previous findings^21,25,33^. However, we report the strongest decrease in alpha and beta power for the medium context constrain conditions. The data contradicts our initial hypothesis and instead indicate that pre-stimulus alpha and beta oscillations are non-monotically related to the amount of contextual constraint which in turn reflects the predictability of the sentence ending. In line with earlier findings, the M/N400 amplitude was monotonically modulated by the degree of constraint, resulting in the lowest amplitude for high, followed by medium and low context constraints. The results suggest that pre-stimulus alpha and beta oscillations and the M/N400 component are neural markers that relate to distinct processes during sentence context evaluation.

In agreement with classic findings, our results show the M/N400 magnitude monotonically decreases with increasing context constraints, and this finding can be taken as support for M/N400 integration and/or predictability accounts^11^. In contrast, the pre-stimulus alpha/beta power fluctuations do not linearly relate to the degree of predictability of the target word given its sentential context. Our findings partially replicate previous results, showing that high context constraints induced a stronger alpha/beta power decrease than low constraint^21,23,25^. Yet, the stronger alpha/beta power decrease for medium context constraints suggest that alpha and beta power does not reflect the degree of predictability of the target word given its context. Furthermore, while we initially defined the alpha frequency band between 8–12 Hz as in previous reports^21,32,38,50^, post-hoc analysis show that the strongest effects of context constraint were observed in the lower alpha range around 8 Hz. This suggests that alpha effects previously linked to sentential constraint processing^25^ may be more prominently observed in the lower alpha range. The interpretation of the results could still be framed within the general mechanistic account of alpha being indicative of neuronal engagement^38^, considering that lower alpha band activity in the 7–9 Hz has been linked to information gating regarding visual processing^51,52^. In addition to the differences in amplitude fluctuations between pre-stimulus alpha and beta oscillations and post-stimulus M400, our effects exhibit distinct topographical properties. The sources of the M400 have previously been localized to temporal as well as prefrontal areas, with a stronger prominence in the left hemisphere (see Lau and colleagues^53^). The topography of the M400 results in the present study is in line with these common findings. In contrast, the sources of the pre-stimulus alpha and beta power changes cover distinct areas. Alpha power modulations were most pronounced over parietal areas, and beta power effects were most pronounced over dorsolateral prefrontal areas. While alpha and beta power reacted similarly to the amount of context constrain within a sentence, the source reconstruction of these effects suggest that alpha power modulations and beta power effects do not reflect the same process. These results capture different alpha and beta dynamics that were reported in studies contrasting low and high sentence context constraints processing only^25,39^. These studies located the alpha and beta power differences predominantly over left inferior frontal areas and left middle and posterior temporal regions.

Pre-stimulus alpha and beta power modulations are not consistent with processes that relate to predictability. However, their dynamics and topographies could speculatively indicate that they relate to attention and/or working memory operations taking place during sentence processing^39^. Alpha oscillations have been previously related to attentional gating and the maintenance of task relevant items in working memory^54–60^. Parietal alpha power is shown to decrease with working memory load during encoding^58,61,62^, and may reflect the relative engagement of networks involved in the encoding of items to keep in memory, and the active inhibition of task irrelevant items^56^. The dorsolateral prefrontal cortex is a crucial region recruited for working memory operations^63–65^. The beta activity reported in our study could potentially reflect working memory load processing capacity^66^. This mechanism is crucial during sentence processing, where pre-stimulus alpha and beta oscillations could be then involved in the preselection and maintenance of lexical candidates^39^ (see also Piai and colleagues^67^). The set of target candidates that need to be retrieved and maintained should in particular be guided by semantic context^68^. We speculate that the amount of possible lexical items to be encoded and maintained might differ depending on the degree of constraint, which would lead to distinct alpha and beta power modulations before target word onset. Compared to low or high context constraining sentences, intermediate context constraints may generate the highest competition between lexical candidates. In the high context constraining conditions, few items are competing, which would require low working memory demands. In low context constraining conditions, the sentential context is broad enough that the number of alternative candidates for sentence ending would be much higher than working memory capacities, resulting in working memory processes being disengaged in this setting. Eventually, in medium context constraining settings, more distractors are to be maintained in working memory than in other context constrains conditions, which in turn is reflected by the stronger alpha power decrease.

As an alternative explanation, the changes in alpha and beta activity could also reflect an overall change of alertness or attentional lapses as a function of the predictability of the stimulus^61,69^. In these studies, participants who had higher alpha power at the presentation of a relevant sentential context were more likely to discard this sentential information for incoming linguistic processing. A decrease in alpha power prior to target word onset could thus potentially reflect general higher levels of alertness that would affect the processing of relevant contextual words. However, we do not think that changes in alertness can explain our results as we controlled our design to result in similar levels of alertness across conditions by randomizing the presentation of the sentences and matching linguistic features like word frequency and length.

In sum, using sentences with different context constraints that are matched on other linguistic variables like lexical frequency and word length, this MEG study shows that pre-stimulus alpha/beta power in the course of the sentence is modulated by context constraints. However, the alpha/beta power decrease is strongest for medium constraining sentences, which defies previous interpretations of this marker in light of a prediction mechanism. The non-monotonic sensitivity of the alpha/beta power fluctuations to these different levels of constraints highlight the importance of including intermediate conditions in language research. Therefore, our results do not support the hypothesis that alpha and beta oscillatory markers reflect linguistic predictability, and a mechanistic account relating alpha/beta oscillations and the degree of sentence context constraint remains to be elaborated.

## Data Availability

The datasets generated during and/or analyzed during the current study are available in the Donders repository, https://data.donders.ru.nl/collections/di/dccn/DSC_3011097.01_968?4.
